# One-Year Readmission Risk and Mortality after Hip Fracture Surgery: A National Population-Based Study in Taiwan

**DOI:** 10.14336/AD.2016.1228

**Published:** 2017-07-21

**Authors:** Tien-Ching Lee, Pei-Shan Ho, Hui-Tzu Lin, Mei-Ling Ho, Hsuan-Ti Huang, Je-Ken Chang

**Affiliations:** ^1^Orthopaedic Research Center, College of Medicine, Kaohsiung Medical University Hospital; ^2^Graduate Institute of Medicine, College of Medicine, Kaohsiung Medical University Hospital; ^3^Department of Orthopaedics, Kaohsiung Medical University Hospital; ^4^Faculty of Dental Hygiene, College of Dental Medicine; ^5^Center of Teaching and Research, Kaohsiung Municipal Ta-Tung Hospital; ^6^Department of Physiology, College of Medicine; ^7^Department of Orthopaedics, College of Medicine, Kaohsiung Medical University Hospital; ^8^Department of Orthopedics, Kaohsiung Municipal Ta-Tung Hospital, Kaohsiung Medical University Hospital, Kaohsiung Medical University, Kaohsiung, Taiwan

**Keywords:** Readmission, hip fractures, mortality, urvival analysis, Taiwanese population

## Abstract

Early readmission following hip fracture (HFx) is associated with high morbidity and mortality. We conducted a survival analysis of patients with readmission within 1 year after HFx to elucidate the trend and predictors for readmission. We used Taiwan National Health Insurance Database to recruit HFx patients who underwent operations between 2000 and 2009. Patients < 60 years; with pathological fractures; involved in major traffic accidents; with previous pelvis, femur, and hip operations; or who died during the index admission were excluded. We used the Chi-square test, logistic regression, Kaplan-Meier method, and Cox proportional hazards model to analyze variables, including age, gender, hospital stay duration, index admission time, and comorbidity on readmission. 5,442 subjects (61.2% female) met the criteria with mean age of 78.8 years. Approximately 15% and 43% HFx patients were readmitted within 30 days (early) and between 30 days and 1 year (late) after discharge, respectively. Highest readmission incidence was observed within the first 30 days. Most common causes of readmission in early and late groups were respiratory system diseases and injuries, respectively. Cox model showed male, old age, hospital stay > 9 days, Charlson Comorbidity Index ≥ 1, index admission during 2000–2003, and internal fixation of HFx were independent predictors of readmission. One-year mortality of the early and the late readmission groups was 44.9% and 32.3%, much higher than overall mortality which was 16.8%. Predictive factors for readmission within 1 year included male, old age, comorbidities, and longer hospital stay. One-year mortality in readmitted patients was significantly higher. HFx patients with these factors need careful follow-up, especially within 30 days after discharge.

Osteoporotic hip fractures cause high mortality and adverse outcomes in the elderly population [1-8]. The annual mortality rate of patients with hip fracture is between 15% and 20% [1, 2, 9]. Previous studies reported that subjects who survived after hip fracture exhibited decreased mobility, a lower quality of life, an increased dependence on family, and an increased requirement for care-givers and social services as well as considerable physical, mental, and financial burdens. Cooper and colleagues estimated that the number of hip fractures worldwide would increase from 1.66 million in 1990 to 6.26 million in 2050, approximately half of which would occur in Asia [1]. Taiwan, along with other developed countries, is facing the challenge of an aging population. In 2011, 10.7% of the entire population was ≥ 65 years; this figure is expected to exceed 14% in 2017[10, 11]. Hip fracture has been a major public health issue. With advances in the quality of medical care, a downward trend from 1999 to 2009 is evident in the annual mortality rate from 18.10% to 13.98% after hip fractures. However, the annual incidence of hip fracture is still increasing from 405/100,000 to 463/100,000[2].

The early readmission rate, generally defined as readmission within 28 or 30 days, is commonly used as a measure of the quality of hospital care [12-15]. Though hospital readmissions are closely related to chronic illness, they are associated with higher morbidity and mortality after hip fractures [16, 17]. Some randomized prospective trials have proved that 12%-75% of readmissions can be prevented [12]. However, there is a lack of information in the literature about the timing and manner of readmission after hip fractures to demonstrate when and how the readmisison occurs.

In this study, we applied a survivial analysis of readmissions within 1 year after hip fracture using the National Health Insurance Research Database (NHIRD) at the National Health Research Institutes (NHRI) in Taiwan to elucidate: 1) the trend of readmission following hip fracture; 2) which factors affect readmission at different time periods; 3) the causes of readmission at different time periods; and 4) whether readmission affects 1-year mortality after hip fracture.

## MATERIALS AND METHODS

### Data sources

The National Health Insurance (NHI) program in Taiwan was implemented in 1995 to provide comprehensive healthcare for all citizens. The program covers all enrolled patients’ medical benefit claims for > 23 million residents in Taiwan, with a coverage rate > 99% of the whole population. The Longitudinal Health Insurance Database (LHID 2000), published by the NHRI in Taiwan, comprises insured population registration files and medical claims for 1,000,000 randomly sampled NHI patients enrolled from the 2000 Registry of NHI Beneficiaries. The random samples have been confirmed to be representative of the population of NHI patients enrolled in Taiwan by the NHRI. The details of database generation are available online (http://nhird.nhri.org.tw/date_cohort.html). This study was exempt from review by the Institutional Review Board because the LHID 2000 consists of de-identified secondary data for research purposes only.

### Study population

We used the discharge codes [International Classification of Diseases, Ninth Revision, Clinical Modification (ICD-9-CM) codes 820-820.9] and the medical codes of internal fixation or hemiarthroplasty (ICD-9-CM codes 79.15, 79.35, and 81.52) in the LHID 2000 to identify hip fracture patients who had undergone operations from January 1, 2000 to December 31, 2009. The admission date of first hospitalization for hip fracture was defined as the index date. The exclusion criteria were inpatients < 60 years, those with malignance associated fractures (ICD-9-CM codes 733.14 and 733.15), those involved in major traffic accidents, or having died during the index admission. The patients who undergone operations on the pelvis, femur, and hip regions before the index date were also excluded to avoid possible confounding effects on complication rate and mortality rate. We recorded the following information: 1) length of hospital stay; 2) operation type: internal fixation (Fix) or arthroplasty (Art); 3) time of admission, divided into three-study periods: 2000-2003, 2004-2006, and 2007-2009; 4) comorbidities retrieved before or at the time of the index date based on the Charlson Comorbidity Index (CCI)[18]; and 5) the main diagnosis code of readmission.

### Outcome measures

This study analyzed three outcomes: 1) the events and causes of readmission within 1 year after index admission; 2) the effects of risk factors on readmission; and 3) mortality within 1 year after index admission.

### Statistical analysis

The Chi-square test and logistic regression were used to compare the patients readmitted within 30 days (early) and between 30 days and 1 year (late) from the date of discharge after the index admission with the patient group without readmission. Time-to-event analysis involved estimating the probability that an event will occur at different points in time. The follow-up endpoint was the date of readmission, and those lost to follow-up were coded by the date of last visit to arrive at “censored” data. The most common time-to-event statistical methods are the Kaplan-Meier method and the Cox proportional hazards model. The Kaplan-Meier estimate was computed to estimate the probability of readmission-free survival. The Cox proportional hazards model was applied to analyze the effects of single and multiple covariates in predicting readmission within 1 year after hip fracture. We compared the effects of variables including age, gender, length of hospital stay, different study period of admission, and number of comorbidities on index admission, using the log-rank test. All analyses were performed using the SAS System (version 9.2; SAS Institute, Cary, NC) and the Statistical Package for the Social Sciences (version 10.0; SPSS Inc, Chicago, IL).

This study complied with the Helsinki Declaration. The data in this study were collected with the approval of the Institutional Review Board of Kaohsiung Medical University Hospital (KMUH-IRB-EXEMPT-20140063) after obtaining informed consent.

**Table 1:** Demographics of Early and Late Readmission Groups.

	Early readmission					Late readmission				

	Yes		No			Yes		No		
Gender	N	%	N	%	P value	N	%	N	%	P value
Female	427	12.82	2905	87.18	<0.0001	1303	39.11	2029	60.89	<0.0001
Male	390	18.49	1719	81.51		1051	49.83	1058	50.17	
Age (years)										
60-69	75	9.42	721	90.58	<0.0001	236	29.65	560	70.35	<0.0001
70-79	318	14.67	1849	85.33		897	41.39	1270	58.61	
≥80	424	17.1	2055	82.9		1221	49.25	1258	50.75	
Time of admission										
2000-2003	305	14.83	1752	85.17	0.0058	891	43.32	1166	81.04	<0.0001
2004-2006	218	13.55	1391	86.45		649	40.34	960	82.91	
2007-2009	201	11.32	1575	88.68		606	34.12	1170	86.09	
Hospital stay										
≤9 days	362	11.72	2728	88.28	<0.0001	1184	38.32	1906	61.68	<0.0001
>9 days	455	19.35	1897	80.65		1170	49.74	1182	50.26	
CCI										
0	298	11.74	2240	88.26	<0.0001	819	32.27	1719	67.73	<0.0001
1	133	17.9	610	82.1		402	54.1	341	45.9	
2	173	16.78	858	83.22		506	49.08	525	50.92	
3	89	18.24	399	81.76		260	53.28	228	46.72	
4+	124	19.31	518	80.69		367	57.17	275	42.83	
Operation										
Art	300	13.24	1966	86.76	0.002	916	40.42	1350	59.58	0.0004
Fix	517	16.28	2659	83.72		1438	45.28	1738	54.72	

CCI, Charlson Comorbidity Index; Art, arthroplasty; Fix, internal fixation

## RESULTS

**Figure 1.**
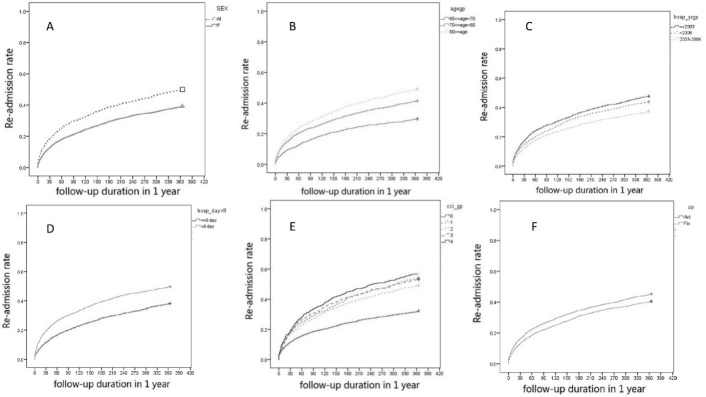
Kaplan-Meier curve showing the time to readmission over the first year following initial discharge after operation for hip fractures **A**) Kaplan-Meier curves by gender (M: male; F: female). **B**) Kaplan-Meier curves by age. **C**) Kaplan-Meier curves by the three-study periods of index admission for hip fractures. **D**) Kaplan-Meier curves by the length of hospital stay. **E**) Kaplan-Meier curves by Charlson Comorbidity Index (CCI). **F**) Kaplan-Meier curves by operation type (Art: Arthroplasty; Fix: Internal fixation).

A total of 5,442 subjects who underwent an operation because of hip fracture met the criteria and were included into the study. The median and mean length of hospital stay during the index admission was 9 days (interquartile range, 7-11 days) and 10.1 days (standard deviation, 6.3 days). Among these patients, 3,333 (61.2%) were female and 2,109 (38.8%) were male, with a mean age of 78.8 years. Of these, 2,266 (41.6%) patients underwent internal fixation and the remaining 3,176 (58.4%) underwent joint replacement; 817 (15.0%) and 2,354 (43.3%) were readmitted within 30 days (early readmission) and between 30 days and 1 year (late readmission) after discharge of index admission, respectively. The average length of index hospital stay was 12.6 days (standard deviation, 9.4 days) in the early readmission group and 11.2 days (standard deviation, 7.7 days) in the late readmission group. The distribution of variables in these two groups is shown in Table 1. The Kaplan-Meier curves (Fig. 1A-1F) reveal the time-to-readmission events within 1 year following hip fractures, with comparisons of different variables including gender, age, length of hospital stay, comorbidity, time of index admission, and type of operation. Analysis revealed that these variables showed similar patterns for time-to-readmission events. A rapid ascending curve was observed in the first 30 days for all patients with hip fracture. According to Figure 1 and Table 1, higher readmission rate was shown in subjects of male gender, of an older age, with the following conditions as length of hospital stay more than nine days, comorbidity (CCI) larger or equal to one, earlier study period of index admission for operation, or with internal fixation of hip fractures at any time over one year after discharge.

### Cox proportional hazards regression model

Further analysis using the Cox proportional hazards regression model was performed (Table 2). In the multiple regression models, we considered the effect of gender, age, length of hospital stay, comorbidities, different study period of index admission, and operation type on readmission. We found that after adjusting for other factors, compared with female patients, male patients had a higher risk of early and late readmission, with a hazard ratio rate (HRR) of 1.46 (1.27, 1.68) and 1.38 (1.27, 1.50) respectively. In addition to male gender, old age, hospital stay > 9 days, comorbidity (CCI ≥ 1), earlier admission, and internal fixation of hip fractures were statistically significant independent risk factors for readmission in both the early and the late readmission groups. Among these variables, old age (> 80 years) was the most powerful risk factor for early readmission, with a hazard ratio rate (HRR) of 1.91 (95% CI, 1.5-2.46). For late readmission, high CCI (≥ 4) was the strongest predictor, with a HRR of 2.12 (95% CI, 1.87-2.4). The patients with hip fractures who were admitted during 2007-2009 carried lower risk of both early and late readmission compared with that during 2000-2003 (HRR of 0.74 and 0.79, respectively).

**Table 2:** Cox Proportional Hazards Regression Model of Early and Late Readmission Groups.

		Early readmission				Late readmission			

		HRR	95%CI		P value	HRR	95%CI		P value
Gender	Female								
	Male	1.46	1.27,	1.68	<0.0001	1.38	1.27,	1.5	<0.0001
Age (years)	60-69								
	70-79	1.61	1.26,	2.08	0.0001	1.5	1.31,	1.74	<0.0001
	≥80	1.91	1.5,	2.46	<0.0001	1.93	1.68,	2.22	<0.0001
Time of admission	2000-2003								
	2004-2006	0.85	0.72,	1.01	0.0601	0.91	0.82,	1	0.053
	2007-2009	0.74	0.63,	0.88	0.0005	0.79	0.71,	0.87	<0.0001
Hospital stay	≤9 days								
	>9 days	1.67	1.45,	1.92	<.0001	1.39	1.28,	1.51	<0.0001
CCI	0								
	1	1.4	1.14,	1.72	0.0016	1.74	1.54,	1.96	<0.0001
	2	1.41	1.17,	1.7	0.0004	1.66	1.48,	1.85	<0.0001
	3	1.45	1.14,	1.83	0.0029	1.76	1.53,	2.03	<0.0001
	4+	1.68	1.35,	2.06	<0.0001	2.12	1.87,	2.4	<0.0001
Operation	Art								
	Fix	1.25	1.08,	1.44	0.0025	1.14	1.05,	1.24	0.0026

CI, confidence interval; CCI, Charlson Comorbidity Index; HRR, Hazard ratio rate; Art, arthroplasty; Fix, internal fixation

### Readmission cause

**Figure 2.**
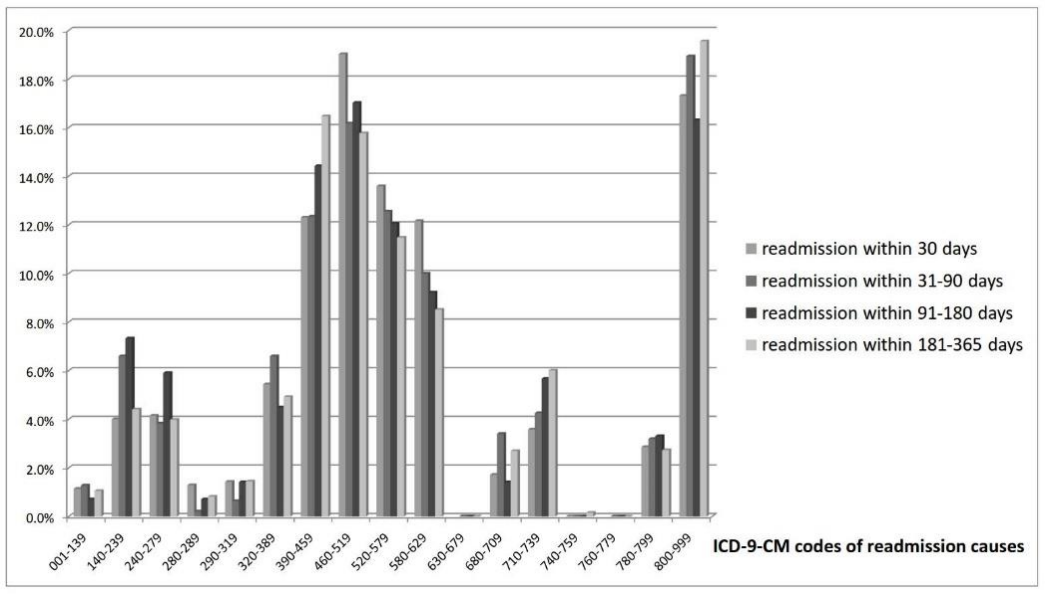
Distribution of readmission causes in different time periods within 1 year using International Classification of Diseases, Version 9, Clinical Modification (ICD-9-CM) coding 001-139: Infectious and parasitic diseases; 140-239: Neoplasms; 240-279: Endocrine, nutritional and metabolic diseases, and immunity disorders; 280-289: Diseases of the blood and blood-forming organs; 290-319: Mental disorders; 320-389: Diseases of the nervous system and sense organs; 390-459: Diseases of the circulatory system; 460-519: Diseases of the respiratory system; 520-579: Diseases of the digestive system; 580-629: Diseases of the genitourinary system; 630-679: Complications of pregnancy, childbirth, and the puerperium; 680-709: Diseases of the skin and subcutaneous tissue; 710-739: Diseases of the musculoskeletal system and connective tissue; 740-759: Congenital anomalies;760-779: Certain conditions originating in the perinatal period.

Figure 2 shows the distribution of readmission diagnoses in different time periods within one year. In early readmission group, the most common causes of readmission were diseases of the respiratory system (19.0%), such as pneumonia, acute respiratory failure, and obstructive chronic bronchitis with acute exacerbation, followed by injuries (17.3%), and diseases of the digestive system (13.6%). In contrast, the most common diagnoses of readmission between 30 days and 1 year (late readmission) are injuries (19.6%), followed by diseases of the respiratory system (16.0%) and diseases of the circulatory system (15.7%).

### 1-year mortality

Table 3 shows the relationship between the time of readmission and the 1-year mortality rate for hip fractures following index admission. The 1-year mortality rates of the early and late readmission groups for hip were 44.9% and 32.3%, respectively. The 1-year mortality rate of the readmission group (within 30, 90, 180, and 365 days) was significantly higher than that of the non-readmission group (*p* < 0.0001) (Table 3).

## DISCUSSION

In this nationwide population-based retrospective study of 5,442 patients who underwent surgery for hip fractures and were discharged from hospital over a period of 1 year, 817 (15.0%) and 2354 (43.3%) were readmitted within 30 days and between 30 days and 1 year following discharge, respectively. The overall readmission rate, calculated using a time-to-event analysis, is similar to those of previous studies, which have reported readmission rates between 11.8% and 34% within 28 or 30 days after discharge [16, 17, 19, 20]. The predictive factors for readmission included male gender, an older age, an earlier period of index admission, a higher number of comorbidities, and a longer hospital stay, in both the early and the late readmission groups. The Kaplan-Meier curves (Fig. 1A-1F) show the time to readmission over the first year following discharge and give the complete picture of how different groups fared over time with respect to readmission [21]. During the first 30 days, a higher risk of readmission was noted. The higher risk observed during the earlier phases of follow-up could be due to postoperative delirium [22], post-hemorrhagic anemia [23], poor appetite [24], and poor general condition. This needs to be verified in further studies.

A number of studies have shown the causes of the early readmission rate after hip fractures [16, 17, 19, 20]. They reported that most early readmissions were because of medical or non-surgical causes such as pneumonia, renal dysfunction, and electrolyte imbalance. In our study, the most common causes of early-readmissions are diseases of respiratory system. It is compatible with previous studies. In contrast to early readmission group, the most common causes of late-readmissions are injuries, such as re-fracture and postoperative mechanical complications.

In the literature, the mean length of the hospital stay in the early readmission group was between 8.5 and 25.4 days [16, 17, 19, 20]. In NHS healthcare-related studies [19, 20], the hospital stay was much longer than that in others [17]. None of these studies revealed that the readmission group had a significantly longer hospital stay during index admission. The reasons for this are multifactorial, including different healthcare policies, payment classification, and different methods of statistical analysis [25]. In this study, the Cox proportional hazards model showed that the length of the hospital stay, where longer than the median number for the entire hip fracture group (9 days) is an independent predictor of readmission (both early and late) in our population. Further studies are needed to elucidate the potential role of hospital stay in readmission rate.

Gender differences in the epidemiology of hip fracture have been reported [26-31], however, there is no previous study assessing the influence of gender on readmission after hip fracture. In the literature, male patients with hip fractures were sicker than the female based on the ASA classification [26-28, 32]. After disregarding the effects of age, operation type, and comorbidity, we still found that males had a higher readmission rate at different time periods than females in Taiwan. We suspect that male patients’ lifestyles and behaviors (e.g., smoking, nutrition, and compliance) may differ from those of females, affecting the prognosis and increasing the readmission rate; however, we have no data to support this speculation.

The type of operation for hip fractures usually depends on the type of fracture. For example, compared with intracapsular fractures, most extracapsular hip fractures (e.g., intertrochanteric fractures) are treated with internal fixation due to good healing potential. However, recent studies have reported that arthroplasty has better outcomes than internal fixation [33-36]. In our study, the higher readmission rate in patients who underwent internal fixation (Table 2) also supports this finding. It may be due to different morbidity profiles of intracapsular and extracapsular hip fracture groups. In comparison with that during 2000-2003, the index admission during 2007-2009 carried lower risk of readmission. A possible explanation includes the improvement of medical and postoperative care and health-related policy with time in Taiwan.

**Table 3:** One-year mortality rate at different time of readmission.

Time of readmission	Mortality within one year				
	Yes		No		
<=30day	N	%	N	%	P value
Yes	325	44.89	399	55.11	P<0.0001
No	587	12.44	4131	87.56	
<=90day					
Yes	495	40.98	713	59.02	P<0.0001
No	417	9.85	3817	90.15	
<=180day					
Yes	605	36.98	1031	63.02	P<0.0001
No	307	8.07	3499	91.93	
<=365day					
Yes	693	32.29	1453	67.71	P<0.0001
No	219	6.64	3077	93.36	

Based on this nationwide population database, the patients with hip fracture who were readmitted within 30 days had three times higher 1-year mortality rate (44.9%) than those that were not (12.4%). This is consistent with the findings of Khan et al. and French et al., who reported an increase in the 1-year mortality rate from 18.7% to 41.8% and 24.9% to 48.5% in their patients who were readmitted within 28 and 30 days, respectively, after discharge following surgery for a hip fracture^17;19^.

There are several limitations to this study. First, the NHIRD was not generated for academic research. Therefore, miscoding and a delay in data processing might have existed. The coding error could be compensated using procedure codes for internal fixation or hemiarthroplasty. Based on the available sample size, the results of this study may have clinically relevant indications and may assist in approaching patients with hip fracture. Second, the NHIRD does not include preoperative joint function/condition, smoking status, severity of comorbidities, patient compliance, nutritional status, biochemical data, socioeconomic characteristics, time to surgery, quality of postoperative care, and falls. Therefore, some unknown confounding factors may exist. Third, we did not include the use and compliance of anti-osteoporotic medication, which may decrease the incidence of secondary hip fracture. Because the follow-up duration in this study was not longer than 1 year, the effect of anti-osteoporotic medication may have been overlooked.

## CONCLUSION

In this population-based study of 5442 patients who underwent surgical treatment for hip fractures, we found the predictive factors for readmission included male gender, older age, a higher number of comorbidities, and a longer hospital stay in both early and late readmission groups. A higher risk of readmission is observed during the first 30 days of follow-up. We suggest that patients with predictive factors need careful follow-up, especially within 30 days following operation for hip fracture.
